# Indigestion Relieved by Food

**Published:** 1879-08

**Authors:** 


INDGESTION RELIEVED BY FOOD.—Dyspepsia and constipation—those twin horrors from which the sedentary brain-workers suffer in America —are invariably relieved, and often entirely overthrown, by the Health Foods. Although employed for a quarter of a century in private practice, by the founder of the Company, it is only five years since these delicate nutriments were first offered to the general public, and in that period more than 30,000 sufferers have found health and comfort in their use. The testimony of their value comes from all sources, and from none more cheerfully and cordially than from physicians. Medical journals have given them favorable mention, and the Cold Blast (Attrition) Whole Wheat Flour received special encomiums at the January (1878) meeting ot the New York State Medical Society, at Albany. All the hospitals now use these Foods to a greater or less extent, and physicians send their patients to the office of the Health Food Company with a food-prescription, just as they were wont to send them to the drug store. Who shall say that this is not a revolution and a reform?

